# Role of T Cell-To-Dendritic Cell Chemoattraction in T Cell Priming Initiation in the Lymph Node: An Agent-Based Modeling Study

**DOI:** 10.3389/fimmu.2019.01289

**Published:** 2019-06-11

**Authors:** Ivan Azarov, Kirill Peskov, Gabriel Helmlinger, Yuri Kosinsky

**Affiliations:** ^1^M&S Decisions, Moscow, Russia; ^2^Computational Oncology Group, I.M. Sechenov First Moscow State Medical University of the Russian Ministry of Health, Moscow, Russia; ^3^Clinical Pharmacology & Safety Sciences, AstraZeneca, Boston, MA, United States

**Keywords:** T cells, dendritic cells, lymph node, chemotaxis, agent-based modeling

## Abstract

The adaptive immune response is initiated in lymph nodes by contact between antigen-bearing dendritic cells (DCs) and antigen-specific T cells. A selected number of naïve T cells that recognize a specific antigen may proliferate into expanded clones, differentiate, and acquire an effector phenotype. Despite growing experimental knowledge, certain mechanistic aspects of T cell behavior in lymph nodes remain poorly understood. Computational modeling approaches may help in addressing such gaps. Here we introduce an agent-based model describing T cell movements and their interactions with DCs, leading to activation and expansion of cognate T cell clones, in a two-dimensional representation of the lymph node paracortex. The primary objective was to test the putative role of T cell chemotaxis toward DCs, and quantitatively assess the impact of chemotaxis with respect to T cell priming efficacy. Firstly, we evaluated whether chemotaxis of naïve T cells toward a nearest DC may accelerate the scanning process, by quantifying, through simulations, the number of unique T cell—DC contact events. We demonstrate that, in the presence of naïve T cell-to-DC chemoattraction, a higher total number of contacts occurs, as compared to a T cell random walk scenario. However, the forming swarm of naïve T cells, as these cells get attracted to the neighborhood of a DC, may then physically restrict access of additional T cells to the DC, leading to an actual decrease in the cumulative number of unique contacts between naïve T cells and DCs. Secondly, we investigated the potential role of chemotaxis in maintaining cognate T cell clone expansion. The time course of cognate T cells number in the system was used as a quantitative characteristic of the expansion. Model-based simulations indicate that inclusion of chemotaxis, which is selective for already activated (but not naïve) antigen-specific T cells, may strongly accelerate the time of immune response occurrence, which subsequently increases the overall amplitude of the T cell clone expansion process.

## Introduction

After maturation in the thymus, immunologically-naïve T lymphocytes (or T cells) continuously circulate between the blood and secondary lymphoid organs, including lymphatic nodes (LNs) and the spleen. Each one of the millions of T cell clones bear unique T cell receptors (TCRs), which define their antigen specificity. In LNs, naïve T cells may encounter dendritic cells (DCs) presenting cognate antigens as MHC-bound peptides (pMHC) on their surface. As a result of such a specific and durable contact T cell-to-DC contact, a naïve T cell may become activated and subsequently proliferate and differentiate into effector forms. This constitutes, in most simplified terms, the essence of the immune response. The fraction of naïve T cells that recognize a particular antigen can be as small as 10^−5^-10^−6^ (1). Since most naïve T cells feature irrelevant specificities, the probability of an immediate contact between a DC bearing a particular antigen and a cognate T cell appears to be very low. Therefore, for efficient antigen recognition, each DC should be in a position to scan a large number of T cells with differing specificities.

Over the past two decades, experiments using two-photon microscopy (2PM) have been applied in the study of murine LNs *in vivo* and have provided a rich set of T cell migration characteristics, as well as information on T cell interactions with antigen-presenting DCs (2). Fibroblastic reticular cells (FRC) form a spatial network throughout the T zone, which is used by DCs as an adhesion scaffold, while T cells use this network as an overall routing system underlying their random migration process. As elucidated from 2PM observations, naïve T cells move with a mean free path of 20–30 μm, interrupted by a change in direction every 2–3 min—a process which, over time, results in a migratory pattern which roughly resembles a “random walk” process (3). During their journey through the LN, naïve T cells are involved in short contacts with DCs, lasting several minutes on average (4–6). DCs migrate slower than T cells, and continuously expand and retract long thin dendrites, thereby significantly increasing the volume of the region they may efficiently scan (6).

Intravital LN observations have shown that cognate T cell interactions with antigen-presenting DCs can be categorized into several stages—with may possibly overlap over time (5, 7): (1) within the first 6 h: transient, serial encounters lasting 10–20 min and upregulation of T cell activation markers; (2) subsequently, and within 14 h: stable binding events lasting for hours, and initiation of cytokine production; (3) consequently, rapid motility followed by short contacts (10–20 min) with DCs, ultimately resulting in T cell proliferation. These observations point to processes of T cells integrating TCR signaling over serial DC contacts, with stage transitions occurring as signal thresholds are being reached. T cell priming in the lymph node spans over 3–4 days, a period after which clonally expanded T cells exit the LN via medullary sinuses (MS) and efferent lymphatics to disseminate in peripheral organs.

Despite such detailed observations, there is no comprehensive understanding, yet, of the detailed mechanisms and dynamics of immune cell interactions; in particular, the fate of individual cells is difficult to track for longer periods of time *in vivo*. As reviewed in (8), methods of computational biology can be used to integrate knowledge, to then simulate cellular dynamics which occur in the LN. In this modeling study, we explored factors influencing specifically the efficiency of T cell repertoire scanning and further expansion of rare cognate T cell clones in a LN. Toward this purpose, we developed a two-dimensional (2D) computational model of T cell–DC interactions and subsequent activation events. In this virtual lymph node, T cell migration, contact dynamics, signal integration and cell division were simulated while computationally tracking the contribution of multiple parameters influencing the properties and functional outcome of T cells, DCs, antigens. In particular, we sought to answer the following questions: (1) May local chemoattraction of naïve T cells toward the nearest DC accelerate the DC scanning process? We chose to quantify this process by modeling the number of unique T cell—DC contact events that occur per time unit, and tracked the evolution of that number over time; (2) May local chemoattraction of activated cognate T cells toward a DC influence T cell expansion efficiency? To this end, the time course of cognate T cell numbers in the virtual LN system was computationally tracked, as a measure of such immune response dynamics.

## Materials and Methods

### Description of the Computational Agent-Based Model

Overall, an agent-based model (ABM) represents a system of interest, with a definition of key players and of relevant interactions among these players that influence the system's behavior. A typical ABM consists of a simulation space (world), stand-alone objects (agents), and rules to set the behavior of individual agents as well as interactions among them (rules). Thus, in our 2D ABM framework, we consider T cell motility and emerging interactions of immune cells within the LN T zone. The T zone was modeled as a lattice of 100 × 100 patches, resulting in an effective physical surface area of 500 × 500 μm^2^ (i.e., 5 × 5 μm^2^ per patch). This modeling framework also considers two types of agents: T cells and antigen-presenting DCs.

**Figure 1:**
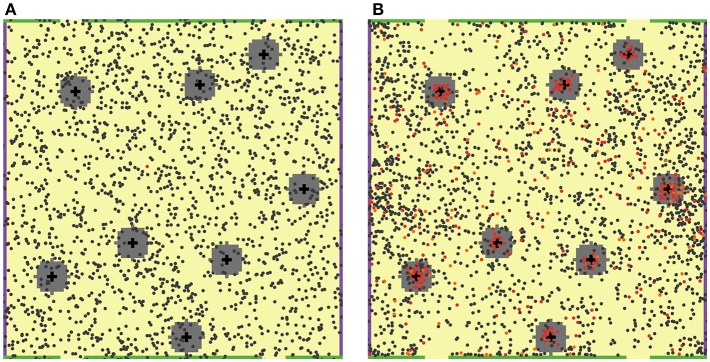
2D ABM model simulation snapshots. T cells are featured as small filled circles of different colors. DCs are shown as larger black cross symbols. “Chemokine cloud” areas are colored in gray, around DCs. Green bars on the top and bottom boundaries of the computational domain physically restrict T cell movement (no escape), while yellow spaces, in between, represent MS structures (T cell escape allowed). **(A)** Snapshot at the beginning of a simulation: 2,000 naïve, non-cognate T cells (dark gray circles) and only one naïve cognate T cell (red circle) are present. **(B)** Snapshot on Day 7 of that simulation: a large number of activated cognate T cells (red circles) proliferated and differentiated into effector T cells (orange circles). The feature of local chemoattraction for activated cognate T cells was turned on in these simulations; consequently, formations of dense swarms of such T cells appear around DCs, as illustrated here.

In order to reproduce interactions between agents present in the LN T zone, 2,000 naïve T cells were randomly placed in this square domain, along with 8 antigen-bearing DCs, randomly placed in 8 fixed positions, as shown in Figure 1. Each 5 × 5 μm^2^ patch was set to contain, at most, one T cell. DCs are typically larger than T cells; thus, it was assumed that a given DC occupies 5 patches, thereby forming cross patterns as shown in Figure 1. Such an initial geometric design would allow each DC to interact with up to 11 neighboring T cells simultaneously.

For simplification, antigen-presenting DCs were assumed to be immobilized, given their migration speed in LNs vs. that of T cells is relatively slow (6), also recognizing that DCs may be more fully anchored onto the reticular network vs. T cells. Once a naïve T cell had found itself in contact with a DC within the neighborhood of a patch, it was allowed to remain in such a state of contact for 3 min (4, 5).

### Definition of T Cell Motion Rules

The stochastic motion of a T cell was implemented in two ways, depending on the particular mechanism tested in the model:

1. ***T Cell Motion via a Random Walk Process:*** In the context of the present 2D ABM model, it was not possible to take explicitly into account interactions of T cells with the FRC network. Hence, empirical rules were introduced to describe T cell motion in this 2D space. To describe the random walk motion process, a T cell was allowed to move, at every discrete time step (30 s) of the simulation, to an unoccupied adjacent position (at a 5 μm distance). This resulted in a modeled T cell velocity of 10 μm/min, in agreement with experimental observations (5).
  To capture the short-term persistence character of T cell movement, T cell motion at each time step was set to be dependent on its previous direction. The new direction was thus calculated by defining a take-off angle from the previous direction, as the sum of two random angles sampled clockwise and counterclockwise from a uniform distribution in the range of (0, θ_max_) degrees. If the resulting adjacent lattice position happened to be already occupied (e.g., by another T cell, or a DC, or a boundary patch on the top or bottom side of the computational domain), then the T cell was computed to remain in its current position, and another direction calculation would be attempted at the next time step.
  In our simulations, we aimed at reproducing not only realistic velocities of T cell movement, but also a physiologically-relevant T cell motility coefficient—realistically reflecting the short-term persistence character of T cell movement. Motility coefficients used in such 2D simulations were calculated from time lapse microscopy records using the formula:
  (1) $$\begin{array}{r} M=\frac{\langle \Delta r^{2}\rangle }{4\tau } \end{array}$$
  where 〈Δ*r*^2^〉 represents the mean squared displacement of an individual T cell from its initial position at time τ. Preliminary simulations were thus performed using different values for θ_max_. Motility coefficient estimates were averaged over 40 T cell trajectories, in each simulation. Simulations with an θ_max_ of 80° resulted in a calculated average motility coefficient of 66 μm^2^/min, which nearly matched experimentally determined estimates of motility coefficients around 68 μm^2^/min (2).
2. ***T Cell Motion Toward a Neighboring DC via a Chemoattraction Process:*** Physiologically, local chemoattraction, or chemotaxis, may be mediated by a concentration gradient of specific chemokine molecules around DCs. In our ABM framework, the effective radius of a “chemokine cloud” around each DC was taken to be 5 patches (~25 μm). T cell motion would switch from a random walk process to a more-or-less directed movement toward the DC center, once inside the “chemokine cloud.” A chemotaxis strength parameter was introduced, representing the probability of performing a directed step instead of a random step. Typically, for simulation purposes, a probability value of 1/3 for a directed step can be used, in agreement with experimental estimates (9).
  After some time spent in the “chemokine cloud,” a T cell loses its sensitivity to the chemokine gradient and starts moving randomly again. The time of T cell de-sensitization to chemokine(s) was taken to be 10 min (10). Such de-sensitization potentially allows a T cell to leave the DC neighborhood. The time for a T cell to get re-sensitized to the chemokine gradient, once outside the cloud, was also assumed to be 10 min.

### Definition of Boundary Conditions

Periodic boundary conditions were applied on the left and right sides of the computational domain: if a T cell were to leave the domain through one side, it would be allowed to immediately re-appear from the other side of the domain, moving in the same direction. In contrast, T cells were not allowed to randomly cross top and bottom boundaries of the computational domain. These boundaries, instead, contained “open patches,” functionally corresponding to medullary sinuses (MS) and efferent lymphatics in a LN. Accordingly, if a T cell were to leave the computational domain through an MS patch at either the top or bottom side, a new T cell would be allowed to enter the computational domain, from a random position through its lateral borders. These settings allowed us to keep an overall constant T cell density in the system under study. The model was explored using a range of numbers of patches containing such MS escape structures (8–120 patches).

### Cognate T Cell Activation and Proliferation

Relatively rare cognate naïve T cells, capable of recognizing DC-presented antigens, were included in the model. No difference in movements, between cognate vs. non-cognate naïve T cells before their first encounter with DC was assumed. After a first encounter, a cognate T cell was set to form a stable contact with a DC for ~24 h; specifically, the duration of contact for each particular cognate T cell was a random value generated from a normal distribution with a mean of 24 h and a variance of 2 h. Upon completion of such a contact, a T cell became activated. The model subsequently simulated activated T cells which randomly migrated into a virtual LN and interacted with DCs bearing cognate antigen complexes. We also explored the option of chemoattraction of activated (but not naïve) T cells toward a neighboring DC.

Similarly to the models by Bogle et al. (11, 12), we assumed that the TCR stimulation signal could be summed over time, during the period of binding and also through sequential DC encounters. Milestones in the activation of a T cell were thus reached when the integrated stimulation were to exceed certain thresholds. In the present model, upon a DC encounter, activated T cells established a contact lasting for about 20 min (a random value generated from a log-normal distribution with a variance of 10 min). T cells were programmed to collect stimulation signals as long as the contact was maintained and to integrate, through summation, such collected stimulation signals upon additional DC encounters. During contact with a DC and the presenting cognate antigen, the stimulation level ***S***of a lymphocyte was set to start increasing to a certain saturation level of a sigmoid curve, according to the following logistic equation:

(2) $$\begin{array}{r} S\left(\text{t}\right)={\text{S}}_{0}+\frac{\alpha }{1\;+{\text{ e}}^{-\beta \text{t}}}, \end{array}$$

where ***S***_**0**_ is the stimulation level at the beginning of the cognate contact. Parameters α = 2.0 and $\beta =0.005\;\overset{-1}{\min}$ values were selected manually, as further detailed in the Supplementary Materials.

For activated T cells which ended up outside a DC contact zone, the stimulation level was set to decrease according to an exponential law:

(3) $$\begin{array}{r} \text{S}\left(\text{t}\right)={\text{S}}_{0}\cdot {\text{e}}^{-\lambda \text{t}}, \end{array}$$

where λ is the exponent indicator, corresponding to a half-life period of 24 h (12), and ***S***_**0**_ is the stimulation level at the start of decay. A typical trajectory of a cognate T cell stimulation signal is shown in Supplementary Figure 2.

Thus, a T cell was set to divide when the stimulation level ***S*** reached a defined threshold. The threshold value of the stimulation level ***S***_***n***_ is one critical model parameter which ultimately affects the proliferation intensity of cognate T cells. We therefore tested multiple threshold values, to estimate the sensitivity of the system to this parameter (Supplementary Figure 3). For the simulations presented here, we selected a value of ***S***_***n***_ = 3.5, which was close to the average ***S***value for all cognate T cells represented in the computational domain.

Two factors were used in the model, to limit the proliferation of activated T cells: (i) a minimal time of about 8 h (random value generated from a normal distribution with a variance of 1 h for each newly formed T cell) was set between successive T cell divisions; (ii) a maximal number of successive proliferating T cell divisions was set. Following the last division of an activated T cell reaching effector status, no further division was allowed, and the cell was eliminated from the system within 24 h. Also, to avoid a strong increase in overall T cell density in the model (as a result of cognate T cell expansion), the following rule was added: if an activated T cell were to leave the computational domain *via* “open patches,” no new T cell was allowed to come back in (in contrast to a naïve T cell leaving). This rule was set to take effect only if the overall number of T cells in the computational domain were to exceed the pre-set “equilibrium” value of 2,000.

### Quantitative Outcomes Simulated via ABM Numerical Experiments

The following quantitative outcome measures were generated:

1. LN transit time: average time from the moment a T cell object appears in the computational domain of the model, until it exits that space through MS patches.
2. Total number of T cell–DC contacts: a cumulative count of contact events between any DC-T cell pair, including possible repeat contacts, as detected during a given simulation time.
3. Calculation of the number of unique T cell–DC contacts: only the first contact of a given T cell with any of the DCs was taken into account. In the model, all DCs were assumed to be strictly identical.
4. Dynamics of cognate T cell number in the computational domain: included naïve, activated and effector T cells, in simulations of up to Day 28.
5. Cumulative outflux of cognate T cells: this was taken as the characteristic measure of the adaptive immune response intensity.

To calculate prediction intervals (90% PI) for each of the model outcomes, 45–100 independent ABM simulations were performed using identical model parameter values, yet different, randomly generated initial T cell and DC positions within the computational domain.

### Software Packages

The *NetLogo 5.0.1* software (13) was used as a computational tool for ABM. Additional details on model development and analysis, e.g., table with parameter values, results of sensitivity analysis and *NetLogo 5.0.1* model scripts are given in the Supplementary Material. The *NetLogo 5.0.1* model code was also uploaded to an open-source repository and is freely available under: https://github.com/Potamophylax/ABM_immune-response/.

## Results

Using the 2D ABM model of a LN as described above, a large number of simulations were performed for various model parameter settings. For simplification purposes in these exploratory simulations, several model parameters were fixed using biologically reasonable estimates. In particular, the assignment of values to T cell and DC densities (respectively, 2,000 and 8 cells per 500 × 500 μm^2^) and to the T cell movement parameter reflecting short-term persistence (θ_max_ = 80°) allowed us to reproduce, *via* simulations, a realistic motility coefficient of T cells in LN.

### Exploration of T Cell Repertoire Scanning Efficacy (Non-cognate Naïve T Cells Only)

Our first goal was to explore the impact of chemoattraction upon efficiency in the process of T cell repertoire scanning, as naïve T cells moved toward a DC. The measure of such efficacy was computed as the rate of accumulation of unique T cell—DC contact events. The chemotaxis strength estimate (*P* = 1/3) was identical to the one used by Riggs et al. (14). A value for an effective radius of the “chemokine cloud” around each DC was selected from the observed size of activated T cell swarms around a DC (~25 μm) (5). Time of T cell de-sensitization and time to T cell re-sensitization (values of 10 min taken for each) reflected characteristic times of cytokine receptor internalization and subsequent recycling.

We specifically explored the model behavior in relationship to the parameter reflective of the size of overall medullary sinuses (MS) and corresponding to the number of “open patches” at the top and bottom boundaries of the computational domain. This parameter is critical in the model, as it regulates T cell turnover rate and thus influences most of the quantitative outcomes. As shown in Supplementary Figure 1, setting the overall MS size in the range of 15–50 patches within the computational domain led to biologically realistic T cell transit times 10–20 h (15).

**Figure 2:**
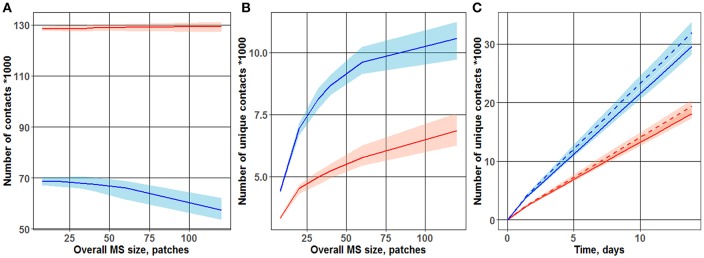
Dependence of the number of total **(A)** and unique **(B)** T cell-DC contacts on overall MS size. Different cases of motion were observed for the two T cell-toward-DC motility scenarios: “random walk” and chemoattraction. Chemotaxis strength ranged from 0 (blue line; “random walk”) to 1/3 (red line). The plot is based on data from 100 simulations, each run over 3 days. Lines represent median values of contact search times; shaded areas represent 90% prediction intervals (PI). **(C)** Accumulation, over time, of unique T cell-DC contacts. Two motility scenarios were used, to describe T cell motion in the vicinity of DCs: “random walk” (two upper curves) *vs*. chemoattraction (two lower dashed curves). Red vs. blue lines reflects different overall MS size values (40 and 32 patches, respectively) used in the simulations.

As shown in Figure 2A, the total number of contacts between T cells and DCs was only minimally affected by the overall MS size. In a “random walk” scenario, it is obvious that the total number of T cell-DC hits depended mainly on the density of T cells within the computational domain, which was kept constant and did not depend on the overall MS size. Interestingly, under a chemoattraction scenario, a much larger total number of contacts was obtained, as compared to a “random walk” motility process (respectively, 130,000 and 70,000 contacts counted during 3 days of simulations). Regions of high densities of naïve T cells (“swarms”) formed locally, around DCs, in simulations under the chemoattraction scenario—which explains this substantial increase in the total number of T cell-DC contacts.

As shown in Figure 2B, the number of unique contacts sharply increased with an increase in overall MS size (which itself is an expression of increased T cell turnover rate through the computational domain). Hence, a higher turnover rate in T cells (from entering to leaving the computational domain) led to a larger number of T cells appearing *de novo* in the computational domain, thereby increasing the probability of new T cells to establish first-time unique contacts with DCs. Conversely, in the case of a slow T cell turnover rate, a larger proportion of T cells may have contacted single DCs multiple times, as they remained for longer times within the computational domain. Simulation results displayed in Figure 2B thus support the following important interpretation: a “random walk” motion scenario for T cells resulted in a substantially higher number of unique contacts between T cells and DCs—a number which is about twice higher *vs*. a chemoattraction scenario. These 2D ABM simulation results are in full agreement with those presented by Riggs et al. (14).

Figure 2C further displays the time evolution of this number of unique T cell-DC contacts, over 14 days of simulations. To explore the influence of the overall MS size upon unique T cell-DC contact dynamics, simulations were performed for two overall MS size values of 32 and 40 patches. The sensitivity of this number of unique T cell-DC contacts with respect to overall MS size, in the considered physiologically-reasonable range, was moderate. The resulting linear dynamics of this number, observed after 1–2 days of simulations, indicated that the system represented by the computational domain reached an equilibrium state by this time. The differing slopes of the two sets of curves on Figure 2C point to different rates of unique T cell-DC contact accumulation; this rate is indeed significantly higher in the case of a “random walk” scenario *vs*. chemoattraction.

### Exploration of Activation and Expansion of Cognate T Cell Clones

A second goal of this study was to explore simulations of cognate T cell priming and expansion under different model parameter settings. Here, we used small, albeit non-zero values for cognate T cell frequency parameters, i.e., the probability of a new incoming T cell to be cognate, which could be activated in contact with DCs and proliferate as described above. Also, we tested different maximal numbers of activated T cell divisions, i.e., maximal numbers of T cell generations starting from naïve cognate T cells to finally differentiated effector T cell. Proceeding from the previous part, we fixed the overall MS size at 32 patches: it ensured a transit time through the LN T zone in agreement with experimental values. All simulations were performed under the assumption of a constant level of antigen stimulation in the LN: neither the number of DCs, nor their positions, nor their properties changed during the simulations.

**Figure 3:**
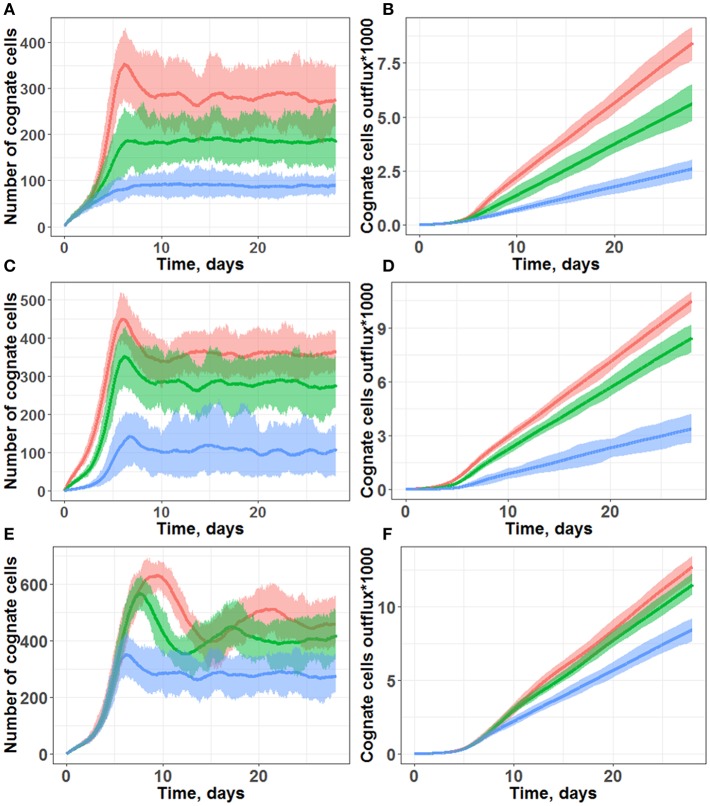
Simulations of cognate T cell numbers in the LN T-zone **(A,C,E)** and their cumulative outflux through efferent lymphatics **(B,D,F)**. **(A,B)** Simulations under scenarios of different chemotaxis probabilities. Multiple chemotaxis strength values were tested: 1/8 (blue line), 1/4 (green line), and 1/3 (red line). The maximal number of divisions was set at 10, and the cognate frequency was set at 1/100. **(C,D)** Simulations under scenarios of different cognate clone frequencies. Multiple cognate frequency values were tested: 1/50 (red line), 1/100 (green line), and 1/500 (blue line). The maximal number of divisions was set at 10, and chemotaxis strength was set at 1/3. **(E,F)** Simulations under scenarios of different maximal numbers of divisions: 10 (blue line), 15 (green line), and 20 divisions (red line). The cognate frequency was set at 1/100, and chemotaxis strength was set at 1/3. All plots are based on measures from 45 simulations lasting 28 days each. Lines represent median values; shaded areas represent 90% PI.

In preliminary simulations not shown here, we sought to reproduce T cell immune responses using the same “random walk” scenario for both naïve and activated lymphocytes; under such conditions, cognate T cell expansion levels were low and not robust. By visual inspection of such simulation trajectories, we observed that most activated cognate T cells would leave the computational domain prior to any cell division occurring. To resolve this technical modeling issue, we allowed chemotaxis toward a neighboring DC to be selective for already activated (but not naïve) cognate T cells. As shown in Figure 3, such an approach allowed us to reproduce realistic T cell immune response dynamics, after varying values of key unknown model parameters over a wide range. Videos S1, S2 (available in the on-line Supplementary Material) illustrate the kinetics of the system at, respectively, the start and Day 7 of representative simulations.

In particular, following days 3–5 of simulations (Figures 3A,C,E), a fast increase in cognate T cells numbers was computed, with distinct peak values around Day 7, in good agreement with the experimentally observed time of 5–7 days for an immune response to occur (16). Beyond 10 days of simulations, all the different trajectories exhibited numbers of cognate T cells which fluctuated around some “steady-state” values, due to a constant level of antigen stimulation which had been assumed in our model. As shown in Figures 3B,D,F simulations, cognate T cell outflux rates also became nearly constant, which correlated with the “steady-state” numbers of cognate T cells within the computational domain.

Model outcomes were highly sensitive to the activated T cell chemotaxis strength value (Figures 3A,B): stronger chemotaxis led to a larger cognate T cell number within the computational domain, *via* a facilitation of T cell proliferation. Taking into account activated T cell chemoattraction led to the accumulation of T cells in forms of swarms around DCs (Figure 1B), which further led to: (a) a longer half-life for these T cells in the LN, slowing down their elimination rate from MS patches; and (b) more frequent contacts with DCs, which favored the build-up of the activation signaling (S) to higher levels. Both factors allowed to effectively increase the number of overall cognate T cell divisions in the system.

Another important factor was the cognate T cell frequency, i.e., the probability for new incoming naïve T cells to recognize antigens presented by DCs (Figures 3C,D). In our simulation framework, a cognate frequency of 1/500 appeared sufficient to induce a robust outflow of cognate T cells (as a main characteristic measure of immune response intensity), however, this outflow rate was twice lower *vs*. a cognate frequency of 1/100. A further increase in the cognate frequency to up to 1/50 only slightly increased the rate of cognate T cell outflow. Additional simulations were performed, over a wide range of cognate frequencies; we determined a non-linear dependence, with a saturation of cognate T cell outflow vs. cognate frequency (see Supplementary Figure 4).

Only moderate increases in “steady-state” cognate T cell numbers and in the corresponding outflows were observed, when the parameter value reflecting the maximal number of T cell divisions was increased from 10 to 20 (Figures 3E,F). However, for a maximal number of divisions ranging from 15 and 20, the model predicted peak values of cognate T cell numbers (within the computational domain) which were twice as high *vs*. when using a maximal number of divisions 10, on Day 10 of the simulations. This complex dynamic behavior reflected in the outcome of cognate T cell numbers was technically traced to a negative feedback loop included in the model, as described in the Methods section. If, during the drive of cognate T cell expansion, the overall T cell density became higher than 2,000 (pre-set T cell “equilibrium” density), the rate of new incoming naïve T cell inflow became smaller. The rate of cognate naïve T cell inflow decreased as well; this, consequently, led to a decrease in the number of cognate T cells within the system. Thus, in the absence of such a negative feedback loop, the domain would become over-populated with T cells, also resulting in “paralysis” of T cell motility.

## Discussion

The original motivation driving this modeling study was to enable the exploration of dynamic spatial effects, in particular a detailed investigation of the relationship between T cell motility behavior and the timing and intensity of an immune response. Intravital microscopy (2PM) yields a wealth of information within a very restricted region of the LN and during a short period of time (hours), whereas histology provides complementary views, yet limited to two dimensions and with no dynamic time element. An ABM of the LN allows for integrative simulations which may help filling the gaps between these two experimental approaches. To explore several hypotheses on T cell motility and their interactions with DCs, we independently developed a simplified, yet biologically reasonable version of a 2D ABM of the LN T cell zone. The model was developed and qualified using *NetLogo* (13), a freely available and flexible software tool.

The 2D ABM presented here, which includes T cell movement, activation and proliferation in a LN, allows for the integration of a number of processes and at the scale of the LN system, and illustrates the necessity to consider all essential processes simultaneously, in order to generate a realistic dynamic picture of the immune response. Because detailed experimental data required to characterize all these processes are not available, we made assumptions regarding several parameters embedded in the model; nonetheless, the model developed here realistically reproduced key temporal characteristics, such as the T cell motility coefficient (2), LN transit times (15) and kinetics of immune response development (16).

Operating characteristics of the model were supported by sensitivity analyses, whereby simulations were run over ranges of model parameter values, with model outcomes being compared against physiological values. Model simulations indicated that the generated T cell response was sensitive to factors, such as naïve cognate T cell frequency and the strength of the hypothetical chemoattraction of T cells toward neighboring DCs. Thus, despite a simplified, semi-empirical structure of the model, we obtained reasonable and robust simulations over a wide range of unknown parameter values.

The ABM LN model presented here was set as a two-dimensional (2D) model, rather than a more physiological three-dimensional (3D) model. A 2D setting of the model allowed us, obviously, to drastically reduce the computational cost of ABM simulations, while carefully estimating the impact of stochastic effects on simulation outcomes. In most of ABMs, T cell movement is implemented as a “random walk” in a non-guided biophysical domain; under such settings, advantages of 3D vs. 2D models may, in fact, not be obvious. The role of the fibroblastic reticular cell (FRC) network in guiding T cell motion in the LN has been studied over many years (17); such a network may adequately constrain T cell movement in a 3D domain. Thus, a realistic spatial structure of the FRC network, together with rules describing lymphocytes and DC interaction within the FRC network should be included in a 3D ABM of the LN. The development of such a detailed, highly parameterized and computationally intensive 3D model is a complex endeavor; the works from a number of such research groups have been reviewed (18). One of the limitations of the present 2D modeling work is the implicit consideration of the FRC network influence, *via* a short-term persistence description of T cell motility, since there would have been no other obvious way to take FRC network effects into account more realistically. In such a 2D context, it should not be expected to reproduce either realistic densities of T cells or realistic numbers of sites of T cells on the DC surface. Thus, the outcomes of our 2D ABM reported here, such as T cells—DC contact numbers and cognate T cell outflux from the LN should be viewed as qualitative measures of behavior, rather than absolute values of cell counts.

The numerical simulations reported here were focused on two pivotal questions. The first question focused on whether local chemoattraction of T cells toward DCs would promote or hamper the scanning efficiency of DCs, within a LN. We demonstrated that, for an effective DC scanning of the T cell repertoire, a T cell “random walk” motility scenario appeared to be the optimal strategy (*vs*. chemoattraction). We provided a physiological rationale, *via* simulations, as to why a chemoattraction motility scenario may actually lead to a non-optimal DC repertoire scanning of T cells: indeed, under chemoattraction, dense and relatively stable swarms of T cells may form around each DC. T cells within these swarms may experience repeated contacts with DCs; and owing to the higher cellular density within swarms, it may take significant time for a T cell to leave a swarm, even if it were to become insensitive to the local chemokine gradient. In addition, swarms may form a barrier for T cells outside the neighborhood, to make contact with DCs. Thus, under a chemoattraction motility scenario, our simulations demonstrated a large number of repeated T cell-DC contacts, while the number of unique T cell-DC contacts, reflective of T cell repertoire scanning efficacy, remained relatively small.

Such results are in full agreement with results from earlier 2D ABM research, in which T cell motility was accurately captured to help determine the impact of chemotactic attraction of T cells-to-DC on repertoire scanning (14). Accordingly, a T cell may move randomly, with a short-term persistence, until it encounters a chemokine gradient around a DC, at which point probabilities are updated so that a T cell is more likely to move toward a DC. Chemokine gradients were captured in a simplified manner, by assigning concentrations in the DC neighborhood (up to 20 μm from the DC). Chemotaxis parameters included strength (related to the likelihood of moving toward a DC), duration (time before de-sensitization occurs), and recovery (time before a T cell may again detect a chemokine gradient). As strength and duration increased, the total number of T cells-to-DC contacts increased, yet the number of unique T cells-to-DC contacts decreased, suggesting that an increased competition of T cells for a DC, resulting from chemotactic-driven movement of T cells toward a DC, interfered with efficient repertoire scanning. In conclusion, a better strategy for efficient scanning is to briefly contact, then clear non-cognate T cells away from an antigen-presenting DC, to make scanning room for different, potentially cognate T cells.

The relevance of a chemo-attraction process on T cell scanning efficiency by DCs was also addressed in modeling work by Vroomans et al. (19), who developed a 2D model of the LN T zone, based on a Cellular Potts Model (CPM) formalism. The CPM is a grid-based spatial model, initially developed to describe the biophysics of cell sorting, based on differential adhesion properties (20). Within this formalism, cell motion is driven by the overall minimization of the energy of deformation and stretching of the cell membrane through stochastic fluctuations, in which global and local forces upon a cell edge are resolved (21). Extension of this CPM approach have been made to describe cell motion under control of a chemokine gradient, including movement under conditions of high cell density in clusters around a DC. In contrast to (14) and our conclusions, model simulations by Vroomans et al. (19) demonstrated that chemo-attraction of T cells does enhance DC scanning efficiency, leading to a greater probability for rare antigen-specific T cells to find DCs bearing the cognate antigen. Also, these authors found that de-sensitization of T cells following contact with a DC would further increase DC scanning efficiency, providing an improvement of nearly 3-fold, vs. a “random walk”-type migration. We here offer one interpretation for this apparent discrepancy: the CPM approach may not adequately reproduce T cell motility in the LN T zone (19). Indeed, in that work, motility was based on a cell adhesion process; also, very dense packing of T cells in the computational domain was assumed. Based on the experimental 2PM observations, a mean free length characteristic of T cell motility was estimated, in the range of 30–40 μm (5, 7). Such a fast, intrinsic velocity of T cell motility would not be possible in the CPM-modeled system (19).

The second question which we sought to address here was about a potential role for chemotaxis in immune response initiation. For such a purpose, we simplified the description of cognate T cell activation, to minimize the overall number of parameters in the model. The concept of a TCR stimulation signal (*S*) accumulation and dynamics of individual cognate T cells was based on previous modeling work (12). However, the implementations of this concept, between the present 2D *vs*. the previously published 3D models were materially different: (a) for simplification, we considered a single cognate T cell clone instead of multiple cognate clones with varying affinities of their TCR to pMHC; and (b) we took into account local chemoattraction of activated cognate T cells toward a DC, as a factor which may accelerate, or even be critical for T cell immune response initiation.

Using our 2D ABM approach, we determined that a feature of selection for activated cognate T cells is required, to reproduce their pronounced expansion upon response to antigen stimulation. As mentioned above, activated T cell chemoattraction lead to T cell accumulation in swarms, around DCs. This effectively caused a longer half-life of these T cells in the LN, slowing down their elimination from MS patches, and also causing frequent contacts with DCs, thereby contributing to activation signaling (*S*) at a higher level. Both factors effectively increased overall numbers of cognate T cell divisions in the LN. The relative contributions of these two factors toward T cell immune response potentiation depended on specific model parameters, such as the activation threshold value (***S***_***n***_), the overall MS size, the T cell motility coefficient, and the overall T cell density. If, indeed, a prolonged half-life of activated T cells in the LN is critical, then an explicit accounting for the effect of sphingosine-1-phosphate receptor down-regulation during T cell activation, leading to retention (for a number of days) of activated T cells in the LN, may be added to the model (22).

Similarly to previous ABM applications tailored to the LN, we showed that such a modeling technique proves to be a useful tool to integrate current knowledge and data on molecular and cellular interactions between immune cells, to then generate novel hypotheses which may guide further experimental studies, to overall improve our mechanistic understanding of the immune activation process that takes place in the LN. Many questions on this dynamical process in the LN remain open, in particular questions on the emerging role of the FRC network in regulating immune responses. Future developments of 3D models, with detailed stromal elements, may play an important role in further elucidating biological mechanisms (18).

## Author Contributions

IA and YK designed, initiated and developed the modeling project. GH and KP participated in discussions and reviewed modeling design and concept. YK and IA wrote the computer program and translated the biological model into agent-based modeling language. IA performed model calculations. GH elaborated on paper outline and points of focus. All authors contributed toward manuscript writing and revisions.

### Conflict of Interest Statement

The authors declare that this study received funding from AstraZeneca. IA, KP, YK are employed by and KP, YK are owners of M&S Decisions, a modeling consultancy which received research funding from AstraZeneca. GH is employed by, and shareholder of AstraZeneca.

## Supplementary Material

Video S1 2D agent-based modelling movie at the start of simulation.

Video S2 2D agent-based modelling movie at the 7th day post numerical experiment start.

Supplementary Information Additional details on the model development and analysis, e.g. table with parameter values, results of sensitivity analysis and additional model simulations.

Source Code NetLogo 5.0.1 scripts corresponding to the final model version presented in the manuscript.
