# Supplementary material for: Role of T Cell-To-Dendritic Cell Chemoattraction in T Cell Priming Initiation in the Lymph Node: An Agent-Based Modeling Study
Source: Front Immunol. 2019 Jun 11;10:1289. doi: 10.3389/fimmu.2019.01289 (PMC6579912; doi:10.3389/fimmu.2019.01289)
Supplement: Supplementary Information — Additional details on the model development and analysis, e.g. table with parameter values, results of sensitivity analysis and additional model simulations. [file Data_Sheet_1.PDF]

Supplementary Material

Role of T Cell-to-Dendritic Cell Chemoattraction in T Cell Priming Initiation in the Lymph Node: An Agent-Based Modeling Study

Ivan Azarov[1], Kirill Peskov[1,2], Gabriel Helmlinger[3], Yuri Kosinsky[1*]

[1]M&S Decisions, Moscow, Russia

[2]Computational oncology group, I.M. Sechenov First Moscow State Medical University of the Russian Ministry of Health, Moscow, Russia

[3]Clinical Pharmacology & Safety Sciences, AstraZeneca, Boston, USA

*** Correspondence:** Yuri Kosinsky: yuri.kosinsky@msdecisions.ru

1 T cell transit and T cell-to-DC contact search times in the lymph node (LN)

As shown in Supplementary Fig. 1, the average transit time of T cells in the computational domain strongly decreased with an increase in the overall size of the medullary sinuses (MS). An increase in MS size may indeed increase the probability for T cells to travel beyond the computational domain. Experimental estimates of T cell transit times in a LN are in the range of 10 to 20 h (1), depending on the cell type (CD4 *vs*. CD8 T lymphocytes) and also on the particular type of LN (mediastinal, hilar, mesenteric, inguinal LN). Thus, setting the overall size of MS in the range of 15-50 patches translated into realistic T cell transit times in our model-based simulations. Also, the choice of a T cell motility regimen (chemo-attraction *vs*. "random walk") had virtually no effect on T cell transit times.

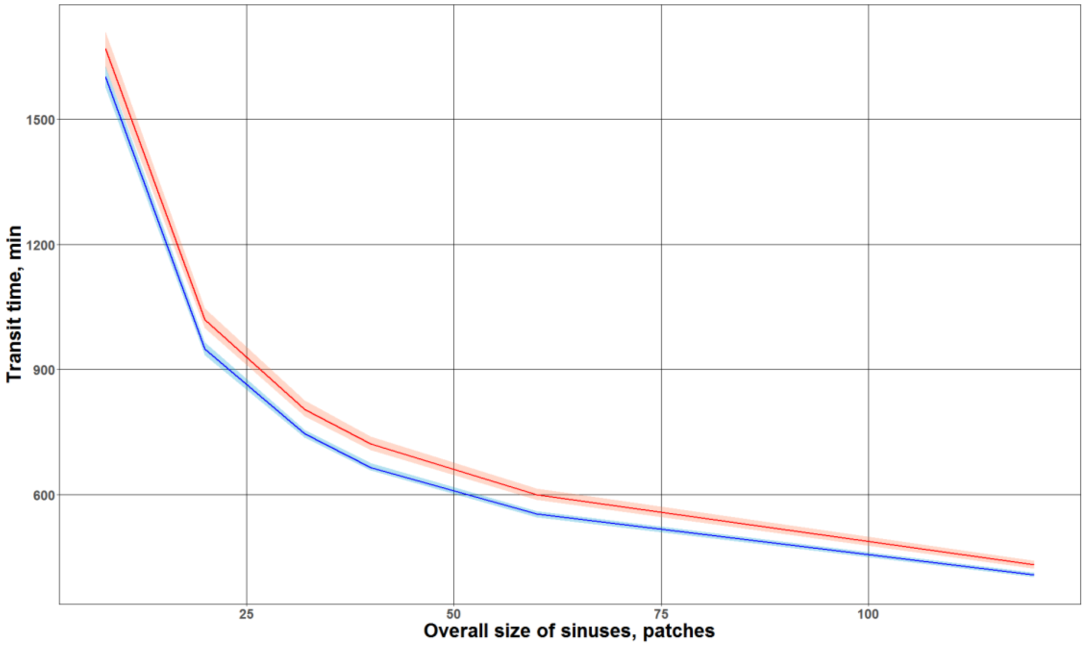

Supplementary Figure 1. Average transit time of T cells through a LN depends on the overall MS size. Chemotaxis strength ranged from 0 (blue line; "random walk") to 1/3 (red line). The plot is based on data from 100 simulations, each run over 3 days. Lines represent median values of transit times; shaded areas represent 90% prediction intervals (PI).

2 Cognate T cell clone expansion model

Activated cognate T cells were programmed according to the model published by Moreau et al. (2): (i) stimulation signal collection, as long as T cell contacts with a DC were maintained; and (ii) integration, through summation, of such stimulation signals upon additional a DC encounter. During contact between a DC and the presenting cognate antigen, the stimulation level ***S*** of a lymphocyte was set to start increasing to a certain saturation level of a sigmoid curve, according to the following logistic equation:

$$\boldsymbol{S(t) = S_0 + \frac{\alpha}{1+e^{-\beta t}}},$$

where $\boldsymbol{S_0}$ is the stimulation level at the beginning of the cognate contact. Parameters $\alpha = 2.0$, $\beta = 0.005\ \text{min}^{-1}$ values were selected manually.

For activated T cells that ended up outside a DC contact zone, the stimulation level was set to decrease according to an exponential law:

$$\mathbf{S(t) = S_0 \cdot e^{-\lambda t}},$$

where λ is the exponent indicator, corresponding to a half-life period of 24 hours (2), and $\boldsymbol{S_0}$ is the stimulation level at the start of decay.

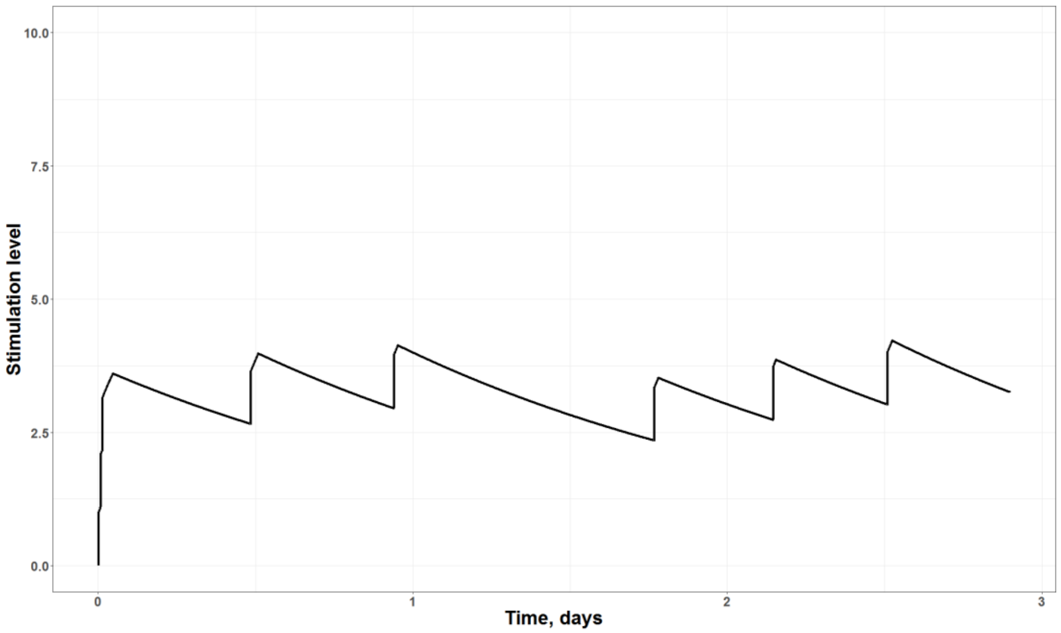

Supplementary Figure 2. An example of a tracked T cell stimulation level (*S*) dynamics. Sharp increases in the stimulation level are associated with the T cell contacting with DCs; declines are associated with natural exponential law decays. For the model parameter setting used, a time-averaged stimulation level of $S \cong 3.5$ was observed.

Values of parameters $\alpha = 2.0$, $\beta = 0.005\ \text{min}^{-1}$ and $\lambda = 0.029\ \text{h}^{-1}$ were selected; the choice of such combined plausible parameter values is neither unique nor based on experimental data, but allowed to adequately reproduce, in simulations, our current understanding of T cell dynamics. We thus fixed these three parameter values, and performed simulations under scenarios of different stimulation level threshold values, above which cell division can occur. As shown in Supplementary Fig.3, the stimulation level threshold value S_n significantly influenced model outcomes dynamics.

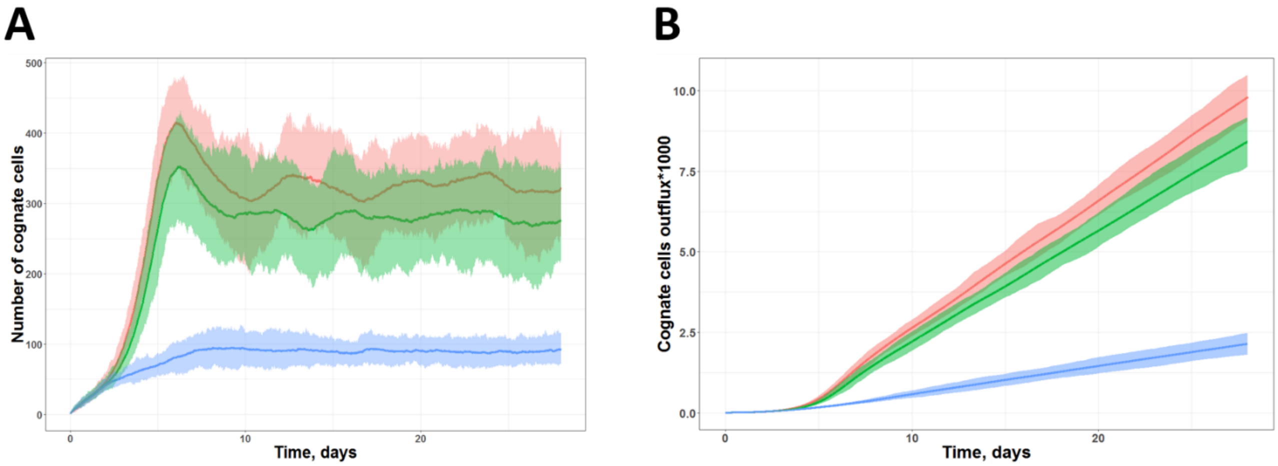

Supplementary Figure 3. Simulations of cognate T cell numbers in the LN T-zone (A) and their cumulative outflux through efferent lymphatics (B), under scenarios of different stimulation level threshold values, above which cell division can occur. The following threshold values, S_n, were tested: 1.0 (red line), 3.5 (green line) and 15 (blue line). The maximal number of divisions was set at 10, the cognate frequency was set at 1/100, and chemotaxis strength was set at 1/3. All plots are based on measures from 45 simulations lasting 28 days each. Lines represent median values; shaded areas represent 90% PI.

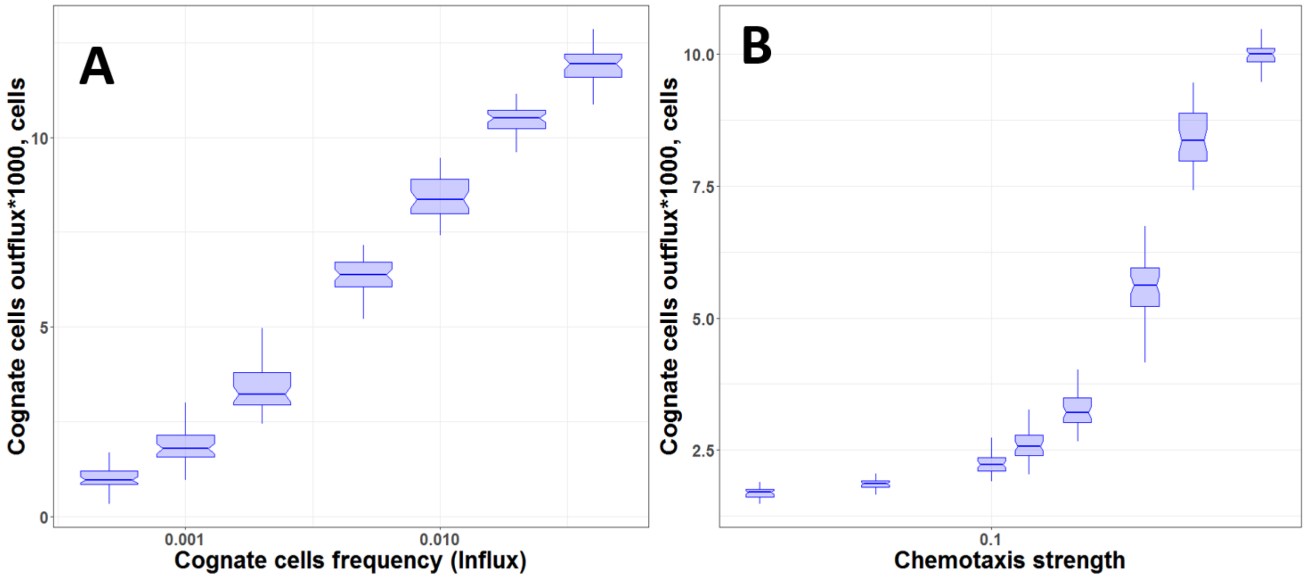

Supplementary Figure 4. Dependencies of cognate T cell cumulative outflux through efferent lymphatics on: (A) cognate T cell frequency, and (B) chemotaxis strength of activated T cells towards a neighboring DC. Cumulative data were taken at the end of the 28-day simulation runs. The following frequencies of cognate clones were used for simulations: 1/25, 1/50, 1/100, 1/200, 1/500, 1/1000, 1/2000 (together with a chemotaxis strength of 1/3 and a maximal number of divisions of 10). The following chemotaxis strength values were tested: 1/40, 1/20, 1/10, 1/8, 1/6, 1/4, 1/3, 1/2 (together with a cognate frequency of 1/100 and a maximal number of divisions of 10).

3 Supplementary Table 1. Summary of model parameters

Parameter	Value	Source
Simulation space size	100 × 100 patches	assumption
Size of patch	5 × 5 μm	assumption
Overall medullary sinuses (MS) size	8, 32, 40, 60, 120 patches	evaluation
Dendritic cell (DC) area	5 patches	[3]
DC number	8	assumption
Initial number of non-cognate T cells	2000	assumption
Initial number of cognate T cells	1	assumption
Discrete time step of simulations	30 s	assumption
T cell velocity	10 μm/min	[5]

θ_{max}, parameter of short-term persistence of T cell movement	80°	evaluation
“Chemokine cloud” radius around each DC	5 patches	[4]
Chemotaxis strength (the probability of performing a directed step towards the DC center instead of a random step)	1/40, 1/20, 1/10, 1/8, 1/6, 1/4, 1/3, 1/2	evaluation and [4]
Time of T cell de-sensitization to chemokine	10 min	[6]
Time of T cell re-sensitization to the chemokine (once outside the “cloud”)	10 min	assumption
Cognate frequency of incoming T cells	0, 1/25, 1/50, 1/100, 1/200, 1/500, 1/1000, 1/2000	evaluation
Non-cognate T cell-DC contact duration	3 min	[7, 8, 9]
Mean of cognate naive T cell-DC contact duration (priming time)	24 h	[9 ,10, 11]
Priming time variance (normal distribution)	2 h	[10, 11]
Mean of activated T cell-DC contact duration	20 min	[7, 12, 13]
Activated T cell-DC contact duration variance (log-normal distribution)	10 min	[7, 12]
S_n, stimulation level threshold	1, 3.5, 15	evaluation
α, stimulation level accumulation parameter	2.0	evaluation
β, stimulation level accumulation parameter	0.005 min^{-1}	evaluation
λ, stimulation level decay rate	0.029 day^{-1}	[2]
Mean time between activated T cell divisions	8 h	[9,14]
Variance of time between activated T cell divisions (normal distribution)	1 h	[9]
Maximal number of proliferating T cell divisions	10, 15, 20	[14]
T effector cell lifespan	60 h	[15]

Supplemental References

1. Mandl JN, Liou R, Klauschen F, Vrisekoop N, Monteiro JP, Yates AJ, Huang AY, Germain RN. Quantification of lymph node transit times reveals differences in antigen surveillance strategies of naïve CD4+ and CD8+ T cells. PNAS (2012) 109:18036-18041. doi:10.1073/pnas.1211717109
2. Moreau HD, Bogle G, Bousso P. A virtual lymph node model to dissect the requirements for T-cell activation by synapses and kinapses. Immunology and Cell Biology (2016) 94:680–688. doi:10.1038/icb.2016.36
3. G.F.Goya, I.Marcos-Campos, R.Fernández-Pacheco, B.Sáez, J.Godino, L.Asín, J.Lambea, P.Tabuenca, J.I.Mayordomo, L.Larrad, M.R.Ibarra, A.Tres (2008). Dendritic cell uptake of iron-based magnetic nanoparticles. *Cell biology international*, *32*(8), 1001-1005. doi:10.1016/j.cellbi.2008.04.001
4. Thomas Riggs, Adrienne Walts, NicolasPerry, Laura Bickle, Jennifer N. Lynch, Amy Myers, Joanne Flynn, Jennifer J.Linderman, Mark J. Miller, Denise E. Kirschner (2008). A comparison of random vs. chemotaxis-driven contacts of T cells with dendritic cells during repertoire scanning. *Journal of theoretical biology*, *250*(4), 732-751. doi:10.1016/j.jtbi.2007.10.015
5. Miller, M. J., Wei, S. H., Parker, I., & Cahalan, M. D. (2002). Two-photon imaging of lymphocyte motility and antigen response in intact lymph node. *Science*, *296*(5574), 1869-1873. doi: 10.1126/science.1070051
6. DeFea, K. A. (2007). Stop that cell! β-Arrestin-dependent chemotaxis: a tale of localized actin assembly and receptor desensitization. *Annu. Rev. Physiol.*, *69*, 535-560. doi:10.1146/annurev.physiol.69.022405.154804
7. Matthias Gunzer, Angelika Schäfer, Stefan Borgmann, Stephan Grabbe, Kurt S.Zänker, Eva-Bettina Bröcker, Eckhart Kämpgen, Peter Friedl (2000). Antigen presentation in extracellular matrix: interactions of T cells with dendritic cells are dynamic, short lived, and sequential. *Immunity*, *13*(3), 323-332. doi:10.1016/S1074-7613(00)00032-7
8. Bousso, P., & Robey, E. (2003). Dynamics of CD8+ T cell priming by dendritic cells in intact lymph nodes. *Nature immunology*, *4*(6), 579. doi:10.1038/ni928
9. Miller, M. J., Safrina, O., Parker, I., & Cahalan, M. D. (2004). Imaging the single cell dynamics of CD4+ T cell activation by dendritic cells in lymph nodes. *Journal of Experimental Medicine*, *200*(7), 847-856. doi:10.1084/jem.20041236
10. Mempel, T. R., Henrickson, S. E., & Von Andrian, U. H. (2004). T-cell priming by dendritic cells in lymph nodes occurs in three distinct phases. *Nature*, *427*(6970), 154. doi:10.1038/nature02238
11. Obst, R. (2015). The timing of T cell priming and cycling. *Frontiers in immunology*, *6*, 563. doi:10.3389/fimmu.2015.00563
12. Friedl, P., & Gunzer, M. (2001). Interaction of T cells with APCs: the serial encounter model. *Trends in immunology*, *22*(4), 187-191. doi:10.1016/S1471-4906(01)01869-5
13. Tobias Rothoeft, Sandra Balkow, Mathias Krummen, Stefan Beissert, Georg Varga, Karin Loser,

Pia Oberbanscheidt, Frank van den Boom, Stephan Grabbe (2006). Structure and duration of contact between dendritic cells and T cells are controlled by T cell activation state. *European journal of immunology*, *36*(12), 3105-3117. doi:10.1002/eji.200636145
14. van Stipdonk, M. J., Lemmens, E. E., & Schoenberger, S. P. (2001). Naive CTLs require a single brief period of antigenic stimulation for clonal expansion and differentiation. *Nature immunology*, *2*(5), 423. doi:10.1002/eji.200636145
15. Sprent, J., & Tough, D. F. (2001). T cell death and memory. *Science*, *293*(5528), 245-248. doi:10.1126/science.1062416
